# Supplementary material for: Validation of a spatial agent-based model for Taenia solium transmission (“CystiAgent”) against a large prospective trial of control strategies in northern Peru
Source: PLoS Negl Trop Dis. 2021 Oct 27;15(10):e0009885. doi: 10.1371/journal.pntd.0009885 (PMC8575314; doi:10.1371/journal.pntd.0009885)
Supplement: S1 Appendix — (DOCX) [file pntd.0009885.s001.docx]

**S1 Appendix. Additional methods for comparison of CystiAgent simulations with observed results from the Ring Strategy Trial**

**Comparison of model outputs with observed outcomes from Ring Strategy Trial**

For each village evaluated, we compared the model-predicted prevalences of human taeniasis, porcine cysticercosis, and porcine seroincidence with corresponding measures observed in the Ring Strategy Trial (RST). Given that the CystiAgent model attempts to represent true infection, and field studies are limited by imperfect diagnostics, we made a variety of adjustments to the observed field statistics in order to compare them with model predictions.

The prevalence of human taeniasis was directly measured at the conclusion of the RST (month 24); however the baseline prevalence was not measured. Therefore, we estimated the baseline prevalence of human taeniasis in RST villages using a log-linear regression equation based on data from prior cross-sectional prevalence study conducted in the same region of northern Peru [1]. In this study, porcine seroprevalence of (2+ EITB bands) was used a predictor for the log-prevalence of human taeniasis, and a regression equation shown below was generated (p = 0.037). For each of the 21 RST villages evaluated during model validation, the baseline prevalence of taeniasis was estimated using this equation and the observed baseline prevalence of porcine seroincidence (Fig A).

$$\log\left( human taeniasis \right)= -5.22+3.19*(porcine seroprevalence)$$

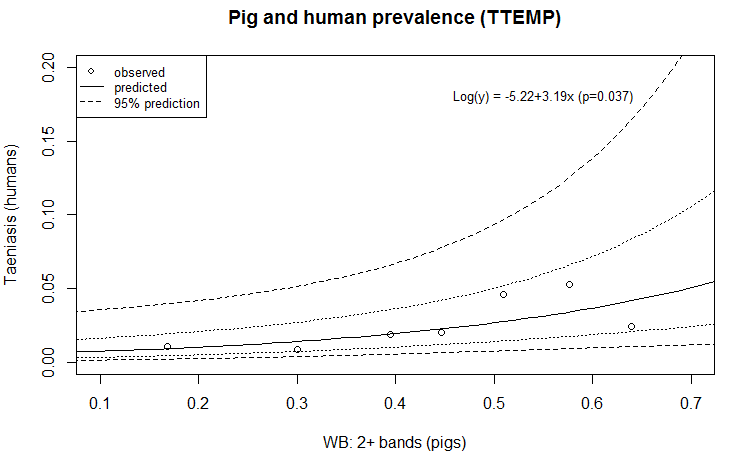


**Fig A.** Regression model used to predict baseline prevalence of human taeniasis from pig seroprevalence. Data were extracted from a cross-sectional survey of seven villages in the Piura region of northern Peru [1].

For baseline and trial-end measurement of porcine cysticercosis, necroscopic examination of pigs was not performed in this study, thus the prevalence of cyst infection at baseline and study-end was estimated based on pig seroprevalence. For both baseline (month 0) and trial-end (month 24) measures, we estimated that 30.1% of seropositive pigs (2+ EITB bands) would have light cyst infection (<100 cysts), and 12.8% of seropositive pigs would have heavy cyst infection (≥100 cysts). For these proportions, we averaged the results from two large necropsy studies conducted in Peru – one in the Piura region [2], and the other in the highland of Huancayo [3]. For seroincdence as a model outcomes, we compared the incidence of pig seroconversion (2+ EITB), which was directly measured in the RST at seven time-points throughout the study with model-predicted seroincidence.

**Participation in interventions**

When available, participation levels for humans and pigs were set separately for each village and each intervention activity. Village-specific participation levels for humans included the proportion of eligible humans that participated in stool screening and presumptive treatment (NSM, 1 vs. 2 doses). Post-screening treatment positively identified humans was fixed at 91.8%, the average observed across all villages. For pigs, village-specific participation levels defined the proportion of eligible pigs receiving anti-helminthic treatment (OFZ) either inside designated treatment rings, or in mass treatment. Pigs were eligible to participate in treatment and/or tongue-screening if they were ≥ 10 weeks old. For repeated interventions, participation levels observed in the field trials were averaged across all rounds for that village.

For ring interventions (ring screening and ring treatment), participation of pigs in tongue screening was set to 77% for all villages and rounds based on observed participation data from RST, and the sensitivity and false-positive rate of tongue screening for detecting heavy cyst infection in pigs was set to 90.9% and 2.1%, respectively [2]. Human participation in the final round mass treatment and post-treatment taeniasis screening applied was set to 73.6% for all villages, based on the observed average in RST villages (Table A).

**Table A. Village-specific participation rates applied to model simulations.**

|  | **Ring Strategy Trial (21 villages)** |
| --- | --- |
| **Humans** |  |
| *Rings* |  |
| Stool screening (CoAg-ELISA) | 83.3% (74.5-89.5) |
| Treatment (NSM) |  |
| 1 dose | 16.1% (12.0-27.0) |
| 2 doses | 70.7% (61.0-76.0) |
| Post-screening treatment | 91.8%^†^ |
| *Mass application* |  |
| Stool screening (CoAg-ELISA) | NA |
| Treatment (NSM, 1 dose) | 75.5% (70.3-81.5) |
| Post-screening treatment | NA |
| **Pigs** |  |
| OXF treatment | 68.9% (26.4-90.0) |

^†^Applied uniformly in all villages

**Drug efficacy and sensitivity of stool screening**

A variety of other intervention settings were applied uniformly to all study villages. The sensitivity of the CoAg-ELISA for detecting *T. solium* taeniasis was set to 96.4% [4], and the efficacy of NSM for treatment of human taeniasis was set at 76.6% for one dose, 86.6% for two doses, and 93.3% for post-screening follow-up. These values were based on results from the screening arms of the Ring Strategy interventions, and are generally in agreement with prior reports of NSM efficacy [5]. Treatment of pigs with OFZ was assumed to have an efficacy of 100% [6] and render cysts non-viable within 1 week [7,8]. For infected pigs, treatment with OFZ conferred protection against future infections for 18 weeks [9].

**References**

1. Pray IW, Ayvar V, Gamboa R, Muro C, Moyano LM, Benavides V, et al. Spatial relationship between Taenia solium tapeworm carriers and necropsy cyst burden in pigs. PLoS Negl Trop Dis. 2017 Apr;11(4):e0005536. PMID: 28406898

2. Flecker RH, Pray IW, Santivaňez SJ, Ayvar V, Gamboa R, Muro C, et al. Assessing ultrasonography as a diagnostic tool for porcine cysticercosis. PLoS Negl Trop Dis. 2017;11(1):e0005282.

3. Gavidia CM, Verastegui MR, Garcia HH, Lopez-Urbina T, Tsang VCW, Pan W, et al. Relationship between serum antibodies and Taenia solium larvae burden in pigs raised in field conditions. PLoS Negl Trop Dis. 2013;7(5):1–8. PMID: 23658848

4. Guezala MC, Rodriguez S, Zamora H, Garcia HH, Gonzalez AE, Tembo A, et al. Development of a species-specific coproantigen ELISA for human Taenia solium taeniasis. Am J Trop Med Hyg. 2009;81(3):433–7. PMID: 19706909

5. Bustos JA, Rodriguez S, Jimenez JA, Moyano LM, Castillo Y, Ayvar V, et al. Detection of Taenia solium taeniasis coproantigen is an early indicator of treatment failure for taeniasis. Clin Vaccine Immunol. 2012 Apr;19(4):570–3. PMID: 22336287

6. Gonzales AE, Garcia HH, Gilman RH, Gavidia CM, Tsang VCW, Bernal T, et al. Effective, single-dose treatment of porcine cysticercosis with oxfendazole. Am J Trop Med Hyg. 1996;54(4):391–4. PMID: 8615453

7. Sikasunge CS, Johansen MV, Willingham AL, Leifsson PS, Phiri IK. Taenia solium porcine cysticercosis: Viability of cysticerci and persistency of antibodies and cysticercal antigens after treatment with oxfendazole. Vet Parasitol. 2008;158(1–2):57–66. PMID: 18834668

8. Gonzalez AE, Falcon N, Gavidia C, Garcia HH, Tsang VCW, Bernal T, et al. Time-response curve of oxfendazole in the treatment of swine cysticercosis. Am J Trop Med Hyg. 1998;59(5):832–6. PMID: 9840607

9. Gonzalez AE, Gavidia C, Falcon N, Bernal T, Verastegui M, Garcia HH, et al. Protection of pigs with cysticercosis from further infections after treatment with oxfendazole. Am J Trop Med Hyg. 2001;65(1):15–8. PMID: 11504400
