# Supplementary figures and images for: Validation of a spatial agent-based model for Taenia solium transmission (“CystiAgent”) against a large prospective trial of control strategies in northern Peru
Source: PLoS Negl Trop Dis. 2021 Oct 27;15(10):e0009885. doi: 10.1371/journal.pntd.0009885 (PMC8575314; doi:10.1371/journal.pntd.0009885)

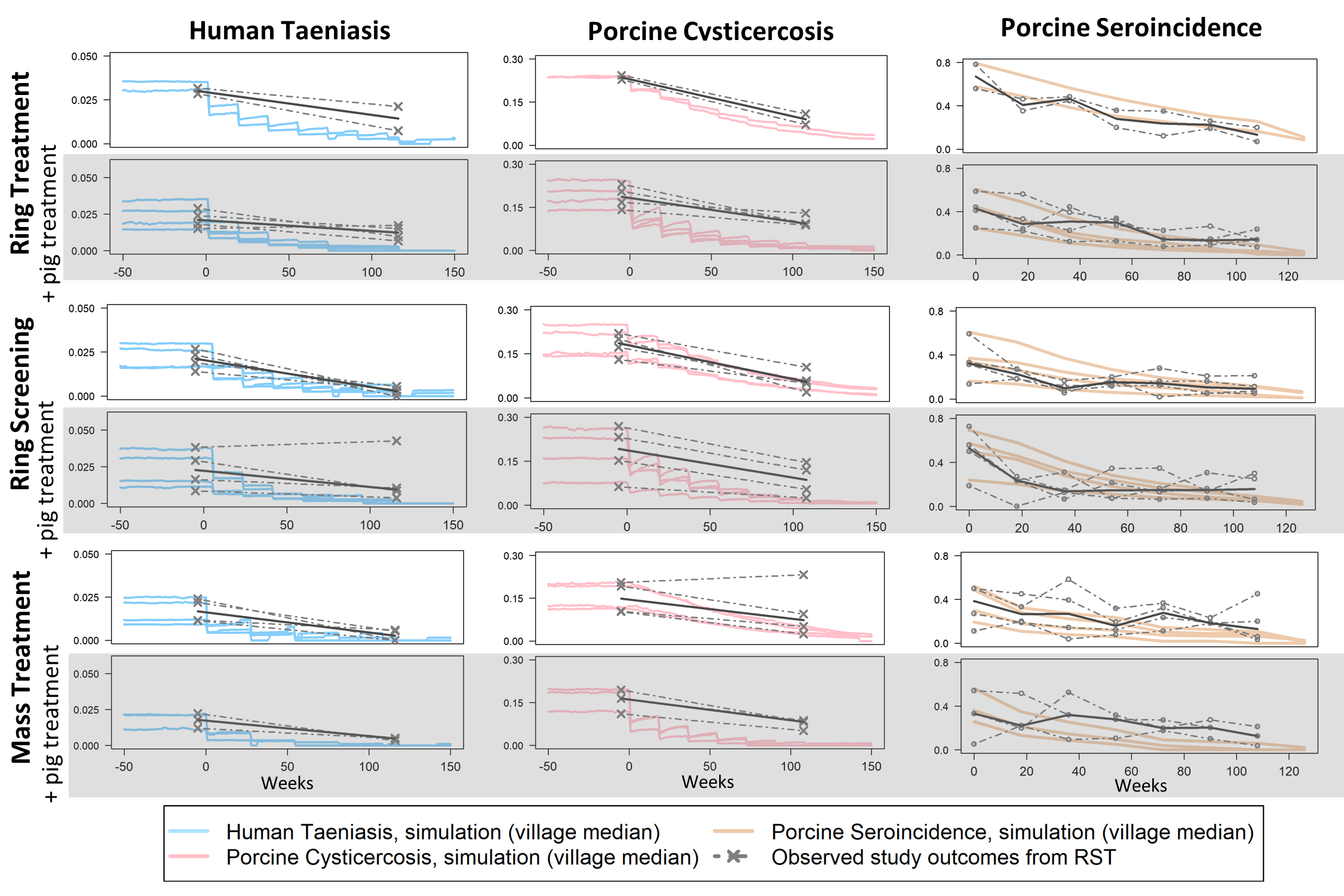

Supplement: S1 Fig — Plots display village-specific median simulated outcomes in CystiAgent across 1000 simulations per village, and observed outcomes from RST (median value within intervention type in bold). For ring treatment (n = 6 villages), ring screening (n = 8 villages) and mass treatment (n = 7 villages villages), interventions with (grey) and without (white) pig treatment are designated by the background color. (TIF) [file pntd.0009885.s003.tif]
