# Supplementary material for: Validation of a spatial agent-based model for Taenia solium transmission (“CystiAgent”) against a large prospective trial of control strategies in northern Peru
Source: PLoS Negl Trop Dis. 2021 Oct 27;15(10):e0009885. doi: 10.1371/journal.pntd.0009885 (PMC8575314; doi:10.1371/journal.pntd.0009885)
Supplement: Attachment — Submitted filename: CystiAgentValidation_ResponseToReviewers.docx [file pntd.0009885.s004.docx]

**Response to Reviewers - "Validation of a spatial agent-based model for Taenia solium transmission (“CystiAgent”) against a large prospective trial of control strategies in northern Peru"**

Dear Editors and Reviewers,

Thank you for your helpful review of our research article, **"Validation of a spatial agent-based model for *Taenia solium* transmission (“CystiAgent”) against a large prospective trial of control strategies in northern Peru."** We have carefully considered your comments and have made revisions to the manuscript in accordance with your suggestions. We have attempted to clarify your questions and concerns to the best of our ability in our responses below, and have made applicable revisions to the manuscript text as requested. We are pleased with your interest in this topic, and are happy to answer any further concerns you may have about the manuscript.

Sincerely,

Ian Pray

Reviewer #1: The manuscript provides a very valuable evaluation of the CystiAgent *Taenia solium* disease transmission model, and generates avenues for further research. I only have some minor suggestions:

1. L418, "prevent.. from accurately replicating observed patterns": one could argue if this is really the goal of any transmission model, or if it would even be possible. To replicate observed patterns, one should have a perfect understanding of reality, while the role of simulation models is rather to better understand reality through simplification. So the lack of a spatially explicit structure, or of an open population structure, would not be a "structural deficit" per se, but rather a design choice, ie, simplification. Thank you, I agree with your comment. These are design features that may be important for modeling, but aren’t necessarily deficits. Text has been changed to: “Prior models have not incorporated certain features of *T. solium* transmission that may be important for model accuracy“.

2. Table 1: it is not always clear how exactly the distribution and parameter should be understood. Do all distributions represent variability, and not uncertainty? For Poisson, Exponential and Binomial (assuming n=1), one parameter would indeed suffice, but for other parameters it would not. I would therefore appreciate some more info on how to interpret the uniform, log-normal and normal distributions. The majority of parameters are proportions that are applied as binomial probabilities in the model (e.g., proportion of households that own pigs is applied as the probability of each house owning a pig), but exponential and Poisson were used for other values. Log-normal and normal distributions also had corresponding standard deviations that were not shown in the original table. I have updated Table 1 to include the standard deviation if applicable, as well as the mean and range of village-specific parameters, and the source data for each parameter.

3. P12L3: the exclusion of 2 villages could be seen as a possible source of bias in the validation results, and could merit some discussion. Does it mean the model is not able to deal with low prevalence settings? Exclusion of these two villages were due to a small pig population (<10 pigs) and the lack of intervention application. Text has been added to the methods and the discussion to address this exclusion.

4. P10L2: Would it be possible to include a reference to the manuscript in review? Yes, coincidentally, we just received news this week that the manuscript was accepted by Emerging Infectious Diseases. While it isn’t yet published, I believe I can now cite this as “in press.”

5. The terms "ring strategy" and "ring strategy trial" are sometimes capitalized, especially in the beginning of the manuscript. The abbreviation "RST" is introduced on page 9 but could be introduced earlier on. Text has been adjusted to define Ring Strategy Trial as RST at first use on the abstract (although the full Ring Strategy Trial was spelled out in section titles and table/figure captions), and un-capitalize “ring strategy” when used descriptively.

6. In the author summary, "T. solium" should be written in full at first occurrence. Done.

Reviewer #2: Intro section:
Minor comments:
1.      Pg. 3 Lines 19-20: presumably the “over 5 million people with epilepsy from NCC” figure comes from Table 1 of Ref[1]; if this is the case, the average figure is 4.85 million cases and relates to persons infected with NCC or epilepsy due to NCC, so this should be written as “approximately 5 million people with neurocysticercosis (NCC) or epilepsy due to NCC”. Done. Thank you.

2.      Pg. 5 Lines 2-3 : This is not correct, existing transmission model with closed population structure would be able to capture rebound in prevalence following intervention cessation (if interventions did not achieve local elimination). The authors would be more accurate in stating existing models would not be able to capture rebound following elimination, due to movement of infected people into an area with successful elimination. Thank you. Text has been adjusted accordingly.

3.      Pg.5 lines 3-5: I do not think the authors can word this so strongly. These deficits *may*result in over-estimation of predictions, but really cross-validation of models on the same datasets will be required to determine differences in predictive capacity (i.e. cross-validation/comparison efforts are more common-place in modelling areas for other NTDs). Thank you. Text has been adjusted accordingly.

=============
Methods section:

1.      It is not clear from the methods text, and tables 1 and Figure 1, how long pigs remain in the “exposed” state, or how this is represented in the model. Does the represent the pre-patent period in pigs (before cyst maturation?)- from Table 1 and description in the methods section text, my impression is that pigs progress immediately after exposure based on the tuning parameter light-inf or heavy-inf without including a cyst maturation period in pigs? Please can the authors make this clearer in the text and describe the way progression from exposed to infected is parameterised in the model. Your interpretation is correct. There was no cyst maturation period included in CystiAgent. This has been clarified in the “Process and Flow” section of the Methods and Figure 1. Despite known cyst maturation period of 2-3 months, this period was not able to be included in the model because pig infection was treated as a categorical state variable – we did not model the maturation of each egg/cyst separately, only the overall infection status of the pig. I don’t suspect this would play a major role in model performance (except perhaps the age-dynamics of pig infections, which were not analyzed in this validation), and is consistent with parameterization of the EpiCyst model.

2.      I am a little unclear regarding the implications of lines 10-13 on page 11; so 42.9% are identified as infected if seropositive (30.1% with light infections plus 12.8% with heavy cyst infections), does this imply that 57.1% are false-positive or antibody positive from the EITB but not harbouring active larval infection (i.e. recovered)? Correct. As detailed in the cited references, EITB is known to be highly sensitive but poorly specific for identifying pigs with active larval infections. The seropositive but uninfected pigs in the model represent those who were exposed to eggs, but did not develop active larval infections (CystiAgent does not incorporate a “recovered” state). The mechanism underlying this process is likely immunological and not detailed in the model. Text has been added to the model description to clarify the lack of a recovered state. Also, we have identified and corrected an error in the reported false positive rate – it now reads 31.1% and 12.8% for a total of 43.9%.

3.      Can the authors indicate whether the copro-antigen ELISA protocol used matched the Guezela et al. 2009 protocl (ref 4 in S1 appendix). (and). This is important as 96.4% sensitivity suggests this protocol was used, whereas sensitivity of copro-antigen ELISA from other literature estimates is generally lower (for example see the modelled estimate from Praet et al. 2013 with a sensitivity of 84.5%). Other copro-antigen protocols based on the original Allan et al. 1999 protocol) give specificity estimates of <100% (not species specific), whereas the Guezela et al. 2009 protocol gave a specificity of 100% (so presumably the authors are assuming 100% specificty, although it is not clear?)- more discussion on the different protocols for copro-antigen ELISA tests can be found in Lightowlers et al. 2016. Thank you. Yes the Guezala et al. method for ELISA was used in the RST. I have clarified this in the appendix, and the main text.

Praet N, Verweij JJ, Mwape KE, Phiri IK, Muma JB, Zulu G, van Lieshout L, Rodriguez-Hidalgo R, Benitez-Ortiz W, Dorny P, Gabriël S. Bayesian modelling to estimate the test characteristics of coprology, coproantigen ELISA and a novel real-time PCR for the diagnosis of taeniasis. Trop Med Int Health. 2013 May;18(5):608-14.

Lightowlers, M W et al. “Monitoring the outcomes of interventions against Taenia solium: options and suggestions.” Parasite immunology vol. 38,3 (2016): 158-69. doi:10.1111/pim.12291

Minor:
1.      Can the authors provide a reference and explain the rationale for the cut-off for light infections with <100 cysts and heavy infections with > 100 cysts (pg.6 lines 6-7). This has been clarified with the following text: “Pig infection is a categorical state variable (susceptible, light infection, heavy infection), and individual *T. solium* eggs or cysts are not agents in the model. Therefore, the cut-off value of 100 cysts between light and heavy cyst infection serves only to allow for translation of prevalence from serological studies [REF] and to fit the conceptual distinction between transmission routes for light and heavy infection, but is not numerically represented in the model. “

2.      Can the authors please state in the table which parameters refer to light-sero and heavy-sero from the description in the text (lines 11-12, page 12) - presumably this is the last row in table 1, but this should be made explicit. Clarified in Table 1.

3.      Please explicitly state that min/max and other quantities are available to specify statistical distributions in the Pray et al. 2020 CystiAgent publication (and that these are the same as presented in this manuscript), as this is not clear. I have updated Table 1 to include the requested information (in response to comments from Reviewer #1 as well). Where applicable (when different parameter values were used for different villages) a mean, min, and max were added. There should no longer be a need to reference Pray 2020 for parameter details, as these are now included in the current table.

============
Results section:

1.      Is there a reason for not estimating the prevalence of cyst infection from the EITB seroincidence data every 4 months, so that prevalence estimates could be more closely compared to simulated dynamics on porcine cysticercosis prevalence throughout the trial (i.e. middle panel in Figure 3)? Equally, why was human taeniasis prevalence not estimated for each timepoint (from the regression model using EITB data) to compare taeniasis prevalence estimates with modelled estimates (throughout the trial)? I think the analysis would be enhanced if these estimates are presented vs. model projections in Figure 3 (rather than just comparing baseline and end-of trial estimates to modelled projections) - the relative/abolsute error plots can still just show differences at baseline vs. end of trial. We agree that the 4-month intervals for model validation against study outcome would allow for a more detailed assessment of model performance. Unfortunately, we were not able to estimate the study outcomes (prevalence of cyst infection or taeniasis) at these time points, only at baseline and study end. This is because in the parent study (RST), we only measured pig seroINCIDENCE at mid-study time point, while pig seroPREVALENCE was measured at baseline and study-end. In other words, we performed a full village-wide seroprevalence survey at beginning and end of the RST study, and during the study only tested new pigs or previously seronegative piglets. Since our calculations for cyst infection and taeniasis were built around estimation from seroprevalence and not incidence, the mid-point incidence estimates would not serve us. That said, we believe that seroincidence is a good indicator for current transmission levels in a community and provides a useful supplemental outcome with which to assess model accuracy at study mid-points.

2.      Where the authors state that various factors are not associated with an improvement or decline in the accuracy of baseline calibration (lines 15-17 pg.14), how was this tested - from the sensitivty analysis in the original Pray et al. 2020 CystiAgent model paper? We performed a multivariable linear regression on the calibration error to evaluate factors that may have been associated with decreased calibration accuracy. The following line was added to the methods sections (“Model Validation”): “After calibration was performed, factors influencing calibration accuracy across villages was assess using multivariable linear regression with relative errors at baseline as the dependent variable.”

3.      The authors state in the legend of Figures 2- 4 that the intervention with and without pig treatment are combined, is there a reason for this? I would recommend showing the model projections and observed data broken down by +/- pig treatment (at least in the supplementary information) to show whether pig treatment has a systematic impact on difference between observed data and projections between ring treatment/ring screening/mass treatment (and the relative error in figure 4).  Thanks for this suggestion, as it was a consideration in the original submission. We chose to combine the interventions (with and without pig treatment) in figures and text descriptions as there wasn’t a noticeable difference in model performance (or intervention effectiveness) between the two sub-arms of each major intervention. That said, we see the value in describing this lack of difference. Thus, Fig 2 has been updated to further sub-divide each of the 3 intervention arms into their two sub-arms. We attempted this in Fig 3, and it created too much visual disorder in the plot to be useful. Therefore, we created a supplemental figure, S3_Figure, that contains each of the sub-plots in figure 3 split out by their study sub-arm. We similarly attempted this in Figure 4, but found that the added lines distracted from the parent-arm comparison (which was the primary objective of Figure 4), thus we did not include this in the re-submission. We have also added some text to further describe sub-arm comparisons I the manuscript.

4.      There seems to be a trial-end point missing for the human taeniasis relative error panel in figure 2 (village 576 for ring screening and village 570 for mass treatment)? Three villages (570, 578, and 585) had no human taeniasis identified at trial-end and relative errors were not able to be calculated. This explanation was added to the figure caption.

Minor:
1.      Is the median porcine seroincidence a % per 4 months (lines 6 on page 14)? Can the authors explicitly state this if so i.e. 44.2% per 4 months at baseline. I recommend in Figure 3 that the seroincidence units on the y-axis (of the 3rd column/panel) should be “proportion per 4 months”. Yes, seroincidence in pigs was a 4-month cumulative incidence. I have clarified this in the text and the figure.

2.      If I am understanding correctly, the median difference referenced on line 8 (for taeniasis) and 11 (porcine cysticercosis) of pg.14  relate to the median (absolute) differences? Can the authors explicitly state whether they refer to the absolute or relative differences throughout the manuscript please. For most measures, we provided both the absolute and relative errors. When absolute errors were presented, we referred to the difference as a unit of percentage points. To further clarify, we have added the word “absolute” throughout the text when applicable.

3.      The abbreviations for porcine cysticercosis (PC) and taeniasis (HT) are first introduced in line 22 on page 15, however should be introduced much earlier, with abbreviations used on line 24 of page 3 (if the authors wish to use abbreviations). Removed. A prior draft used abbreviations throughout, and it appears we missed one. Thank you.

4.      On line 17-18 of page 16, should this read as range: -36.2 to +18.7% percentage points or 36.2 lower to 18.7% higher to improve clarity. Done.

==============
Discussion section:

1.      The authors indicate that they have used data to suggest that 44% of EITB-seropositive pigs would be infected (should this not be 43% from 30.1% light infected + 12.8% heavy infected?) – I think an improved approach would be to adjust observed prevalence data/estimates to 'informed' or 'true' prevalence with the methodology proposed by Speybroeck et al. 2013 (using a Bayesian framework, incorporating uncertainty in diagnostic sensitivity and specificity)? If evidence from previous intervention programmes suggest that the false positive rate (specificity) changes (as the authors suggest in lines 1-3 of page 21), the observed results in the RST could be adjusted based on the method proposed by Spebroeck et al. 2013 with varying specificity throughout the programme? I recommend this as an area for the authors to consider for adjusting their field data, especially if the diagnostic parameters change throughout intervention (which, as the authors suggest may change the error
between simulated prevalence and prevalence estimates from the data).

First, there was a typo in the number. It has been changed to 31.1% and 12.8%, which is 44%.

Second, thank you for the suggestion to use a Bayesian approach. Indeed, estimating the final-round pig prevalence in this study was challenging and perhaps one of the most impactful decisions on our conclusions for model accuracy. There is considerable uncertainty in the performance of EITB serology and few studies have been conducted to assess their performance under normal endemic conditions (among them are the two we reference), let alone after a control intervention has been applied (none that I’m aware of). Based on my reading of the referenced paper, it appears that this Bayesian approach could allow for estimation of final-round prevalence using different sensitivity and specificity values at baseline versus final, which could improve the flexibility of our analysis and the model performance measures. But if I’m not mistaken, this approach still requires at least some basis by which to estimate (or project) what the new sensitivity and specificity would be in these new conditions (post intervention). Our challenge is that there is no available data on which to base such a projection. We have only hypothesized that the test performance could change under post-intervention conditions, but don’t know that it would. In the discussion, we reference the seminal work by Garcia et al (Elimination of T. solium in Northern Peru) which did demonstrate high seropositivity and low necropsy-positivity in pigs following an elimination program, but this was not a formal evaluation of diagnostic performance and was performed in a different region under different circumstances, thus we chose not to use this to formally estimate porcine cysticercosis prevalence. I have edited the text in this section of the discussion to highlight the uncertainty and reason for not using these data.

Because of this, we would prefer to retain our original calculation of study-end prevalence, as having been calculated with the best available data, and strongly highlight the potential differences in diagnostic performance as a potential limitation and opportunity for future work. We appreciate your insight and questions and hope that this is a satisfactory resolution.

Speybroeck N, Devleesschauwer B, Joseph L, Berkvens D. Misclassification errors in prevalence estimation: Bayesian handling with care. Int J Public Health. 2013 Oct;58(5):791-5.

Minor:
1.      Could the authors explain the statement that the number of pigs with acquired immunity would decrease as control interventions are applied (lines 9-10 on page 19); is this because reduction in environmental contamination of the environment (through treatment of taeniasis carriers) would result in lower exposure of pigs (and therefore development of immunity)? Does this then assume that susceptibility in the pig population is higher when pigs are subsequently exposed (following imported cases of human taeniasis carriers moving into the area?) I think the authors could expand on this point further. Yes, we hypothesize that immunity would decrease as control pressure is applied due to pigs having less frequent exposure to *T. solium* eggs in the environment. When transmission is high in endemic areas, we assume that most pigs (particularly free-roaming pigs) have high levels of exposure, some will develop infections, some will clear infections and remain immune for a period of time, but the prevalence is relative stable with few truly susceptible pigs. After treatment applications, many pigs are susceptible again, and a single tapeworm carrier introduction could lead to a steep rebound. The impact on our current validation work is that without modeling immunity specifically, we underestimate the magnitude of the rebound effect, and overestimate the intervention effect. This could mean that transmission rebound may happen more quickly in a population post-intervention. I have added some clarification to this in the main text.

--------------------
