# Supplementary material for: Validation of a spatial agent-based model for Taenia solium transmission (“CystiAgent”) against a large prospective trial of control strategies in northern Peru
Source: PLoS Negl Trop Dis. 2021 Oct 27;15(10):e0009885. doi: 10.1371/journal.pntd.0009885 (PMC8575314; doi:10.1371/journal.pntd.0009885)
Supplement: Attachment — Submitted filename: CystiAgentValidation_PLOS_ResponseToReviewers.docx [file pntd.0009885.s005.docx]

**Response to Reviewers**

Reviewer #2: Thank you for you responses, they certainly helped and the improvements to the manuscript are noticeable. I have a few remaining questions to follow-up:

Original Q.1: It is not clear from the methods text, and tables 1 and Figure 1, how long pigs remain in the
“exposed” state, or how this is represented in the model. Does the represent the pre-patent period in pigs (before cyst maturation?)- from Table 1 and description in the methods section text, my impression is that pigs progress immediately after exposure based on the tuning parameter light-inf or heavy-inf without including a cyst maturation period in pigs? Please can the authors make this clearer in the text and describe the way progression from exposed to infected is parameterised in the model.

**New question:
Thanks for this. I think the section could do with a little more explanation/clarification, as an exposed compartment (as seen in Fig 1) would normally refer to individuals exposed but not infectious (in this context egg establishment and cyst maturation to a mature cyst, governed by a rate of transition). But I understand the exposed compartment, within this agent-based model formulation, represents an instantaneous transition governed by the light-all or heavy-all stochastic tuning (probability of exposure) parameters, and then an instantaneous transition to light infection (based on the probability of light infection upon contact parameter light-inf), or to heavy infection (based on the probability of heavy infection upon contact parameter heavy-inf). To this end, I think Figure 1 should be adjusted to provide more clarity on the transmission pathways: for example I think, if my understanding is correct, that there should be two pig exposure compartments, with light-all
and heavy-all parameters to Exposed_light (EL) and Exposed_heavy (EH), which independently lead to the IL or IH compartments (and then two transitions from for EL and EH to IS based on light-sero and heavy-sero tuning parameters, which will improve clarity (if my understanding here is correct).**

**Furthermore, I think Figure 1 could be improved by detailing the tuning parameters between compartment transitions, so that Figure 1 and Table 1 align more closely (also please see Figure 2 in the CystiSim Braae et et al. 2016 paper for example model schematic including transition probabilities within an IBM).**

**RESPONSE:** Thank you for this suggestion. Your understanding of the exposure compartments and division between EL and EH in CystiAgent is correct, and had not been previously depicted in Figure 1. I have updated figure 1 to include 2 panels – panel A has the overall flow of processes (original) and panel B has the more detailed breakdown of SEIR compartments as suggested. I’ve also included the tuning parameters into panel B, as suggested.


In response to question & answer (3):

Original Q.3: I am a little unclear regarding the implications of lines 10-13 on page 11; so 42.9% are identified as infected if seropositive (30.1% with light infections plus 12.8% with heavy cyst infections), does this imply that 57.1% are false-positive or antibody positive from the EITB but not harbouring active larval infection (i.e. recovered)?

**New question: Thanks for this clarification, I think some text here with the relevant reference to briefly outline that the EITB is highly sensitive but not highly specific (hence the high false positive proportion due to measuring pigs that are exposed rather than only infected), would be useful in the first paragraph after Trial Outcomes (and to put the 56.1% FP proportion into context).**

RESPONSE: The following text was added to the paragraph to justify and explain the conversion from serology to cyst infection: EITB serology is highly sensitive but non-specific for identifying pigs with active larval cyst infection (seropositivity without infection may be due to prior resolved cyst infection, exposure without established infection, or cross-reactivity), thus conversion is needed account for EITB positive pigs without active cyst infections [33-35].

Minor:
Original Q:Can the authors provide a reference and explain the rationale for the cut-off for light infections
with <100 cysts and heavy infections with > 100 cysts (pg.6 lines 6-7).

**New question: Thanks, I think the authors may have missed a reference here in the updated text after “Exposure to eggs may cause light cyst infection (<100 cysts), and exposure to proglottid segments may lead to heavy cyst infection (≥ 100 cysts); either may lead to seropositivity” if this cut-off is based on data, or if not please state whether this is an assumption based on expert-opinion?**

RESPONSE: Thank you. I’ve added 3 references that are cited elsewhere here for the cut-off between 100+ and <100 cysts for heavy and light cyst infection. As you know, the actual distinction between +/-100 is arbitrary, and is being used as a general construct for the different pathway (egg vs. proglottid). Here we needed to use an exact number to allow calculation based on the prior necropsy studies, which used 100 cut-offs. I added references to the necropsy studies here and the following text as justification:

The different exposure-infection pathways for heavy and light cyst infection in pigs were based on a combination of biologic plausibility and consistency with prior transmission models [18,19].

--------------------

<b>Results</b></br></br>
-Does the analysis presented match the analysis plan?</br>
-Are the results clearly and completely presented?</br>
-Are the figures (Tables, Images) of sufficient quality for clarity?</br></br>

Reviewer #1: (No Response)

Reviewer #2: Again, thank you for your responses which were very clear, and the corresponding edits to the manuscript. I have one remaining question:

Original Q. If I am understanding correctly, the median difference referenced on line 8 (for taeniasis) and 11 (porcine cysticercosis) of pg.14 relate to the median (absolute) differences? Can the authors explicitly state whether they refer to the absolute or relative differences throughout the manuscript please.

**New question: Thanks for the amendments and clarifications. I am not sure I fully understand the axis and accompanying results text for Figure 2. Does the x-axis on figure 2 for absolute error (bottom two panels) refer to (% points difference), as I am not sure how a median absolute error (difference) is therefore estimated from Figure 2 (i.e., the absolute error difference at baseline for taeniasis seems to have a max lower limit across villages of -0.03 and max upper limit across villages of +0.065)?.**

RESPONSE: I have clarified in the figure legend that the x-axis is in absolute decimals, not percentage point. To avoid confusion with the percentage difference presented in the relative errors, we chose to present the exact decimal differences in the absolute error figure. However, to facilitate easier interpretation in the main text, we have presented the absolute errors as percentage points. To see how these ranges relate to numbers presented in the text, the figures would be multiplied by 100. Hopefully, doing this will allow for the calculations you worked out above to be clearer (I don’t exactly follow the numbers you’ve listed). The absolute error for taeniasis at baseline is very small with a range of -0.002 (or -0.2 percentage points) to +0.0063, or 0.63 percentage points). This is difficult to distinguish in the graph as all of the dots line up closely with the 0 line.

--------------------

<b>Conclusions</b></br></br>
-Are the conclusions supported by the data presented?</br>
-Are the limitations of analysis clearly described?</br>
-Do the authors discuss how these data can be helpful to advance our understanding of the topic under study?</br>
-Is public health relevance addressed?</br></br>

Reviewer #1: (No Response)

Reviewer #2: (No Response)

--------------------

<b>Editorial and Data Presentation Modifications?</b></br><br/>
Use  this section for editorial suggestions as well as relatively minor modifications of existing data that would enhance clarity. If the only modifications needed are minor and/or editorial, you may wish to recommend “Minor Revision” or “Accept”.

Reviewer #1: (No Response)

Reviewer #2: (No Response)

--------------------

<b>Summary and General Comments</b></br></br>
Use  this section to provide overall comments, discuss strengths/weaknesses of the study, novelty, significance, general execution and scholarship. You may also include additional comments for the author, including concerns about dual publication, research ethics, or publication ethics. If requesting major revision, please articulate the new experiments that are needed.

Reviewer #1: Thank you for addressing my comments.

Reviewer #2: (No Response)

--------------------

PLOS authors have the option to publish the peer review history of their article (<a href="<https://journals.plos.org/plosntds/s/editorial-and-peer-review-process#loc-peer-review-history>" target="_blank">what does this mean?</a>). If published, this will include your full peer review and any attached files.<br><br>

If you choose “no”, your identity will remain anonymous but your review may still be made public.<br><br>

<b>Do you want your identity to be public for this peer review?</b> For information about this choice, including consent withdrawal, please see our <a href="<https://www.plos.org/privacy-policy>" target="_blank">Privacy Policy</a>.

Reviewer #1: Yes: Brecht Devleesschauwer

Reviewer #2: Yes: Matthew Dixon-Zegeye
